# Aberrant Monoaminergic System in Thyroid Hormone Receptor-β Deficient Mice as a Model of Attention-Deficit/Hyperactivity Disorder

**DOI:** 10.1093/ijnp/pyv004

**Published:** 2015-03-20

**Authors:** Masanori Ookubo, Miyuki Sadamatsu, Atsushi Yoshimura, Satoru Suzuki, Nobumasa Kato, Hideto Kojima, Naoto Yamada, Hirohiko Kanai

**Affiliations:** Department of Psychiatry, Shiga University of Medical Science, Shiga, Japan (Drs Ookubo, Yoshimura, Yamada, and Kanai); Department of Psychiatry, Minakuchi Hospital, Shiga, Japan (Dr Ookubo); Department of Psychology and Psychiatry, Human Sciences, Kinjo Gakuin University, Aich, Japan (Dr Sadamatsu); Department of Thyroid and Endocrinology, Fukushima Medical University, Fukushima, Japan (Dr Suzuki); Department of Psychiatry, Showa University School of Medicine, Tokyo, Japan (Dr Kato); Department of Molecular Genetics in Medicine, Shiga University of Medical Science, Shiga, Japan (Dr. Kojima); Department of Psychiatry, Japanese Red Cross Society Nagahama Hospital, Shiga, Japan (Dr. Kanai).

**Keywords:** ADHD, histone deacetylase, serotonin, dopamine, reward system

## Abstract

**Background:**

Thyroid hormone receptors are divided into 2 functional types: TRα and TRβ. Thyroid hormone receptors play pivotal roles in the developing brain, and disruption of thyroid hormone receptors can produce permanent behavioral abnormality in animal models and humans.

**Methods:**

Here we examined behavioralchanges, regional monoamine metabolism, and expression of epigenetic modulatory proteins, including acetylated histone H3 and histone deacetylase, in the developing brain of TRα-disrupted (TRα^*0/0*^) and TRβ-deficient (TRβ^*−/−*^) mice. Tissue concentrations of dopamine, serotonin (5-hydroxytryptamine) and their metabolites in the mesocorticolimbic pathway were measured.

**Results:**

TRβ^*−/−*^ mice, a model of attention-deficit/hyperactivity disorder, showed significantly high exploratory activity and reduced habituation, whereas TRα^*0/0*^ mice showed normal exploratory activity. The biochemical profiles of dopamine and 5-hydroxytryptamine showed significantly low dopamine metabolic rates in the caudate putamen and nucleus accumbens and overall low 5-hydroxytryptamine metabolic rates in TRβ^*−/−*^ mice, but not in TRα^*0/0*^ mice. Furthermore, the expression of acetylated histone H3 was low in the dorsal raphe of TRβ^*−/−*^ mice, and histone deacetylase 2/3 proteins were widely increased in the mesolimbic system.

**Conclusions:**

These findings suggest that TRβ deficiency causes dysfunction of the monoaminergic system, accompanied by epigenetic disruption during the brain maturation process.

## Introduction

Sufficient amounts of thyroid hormones are produced after birth, and the expression of thyroid hormone receptors (TRs) in the brain of rodents has been shown to peak during the neonatal period (Bradley et al., 1992). TRs play multiple roles in the structural and functional development of the immature brain, including neuronal cell maturation, migration, and synaptogenesis as well as glial proliferation/maturation (Cheng et al., 2010). Triiodothyronine functions are mediated by 2 major TR isoforms: TRα and TRβ, which are differentially expressed in cell types and brain regions to control the expression of a wide array of genes during development (Bradley et al., 1992; O’Shea and Williams, 2002). TRα expression is widely distributed in the brain, whereas TRβ transcript expression is more transient and restricted in distribution (Bradley et al., 1989, 1992). Such a spatiotemporal expression of TRs is presumed to have a complicated contribution to the establishment of functions of the mature brain.

Resistance to thyroid hormone is an inherited syndrome caused by a mutated TRβ gene with a reduction or loss of triiodothyronine binding activity or the transcriptional capacity (Hauser et al., 1993; Brucker-Davis et al., 1995). Of note, there is a high proportion (up to 60%) of patients with resistance to thyroid hormone syndrome who have the comorbidity of attention-deficit/hyperactivity disorder (ADHD) (Hauser et al., 1993; Brucker-Davis et al., 1995), although the majority of these patients are heterozygous. On the other hand, TRα gene disruption is rare, and there is quite limited information on its association with mental illness (Schoenmakers et al., 2013).

ADHD is characterized by hyperactivity, impaired sustained attention, impulsivity, and distractibility; it affects 3 to 7% of the school-age population worldwide and is the most diagnosed disorder in children (Weyandt and Dupaul, 2008). Children with ADHD are clinically heterogeneous and are classified into 3 subtypes: predominantly inattentive, predominantly hyperactive-impulsive, and the combined type. Thyroid hormone disruption during pregnancy is presumed to be a risk factor for ADHD, including iodine deficiency (Vermiglio et al., 2004) and exposure to bisphenol A, a food contaminant chemical that acts as a thyroid hormone disruptor (Xu et al., 2007; Harley et al., 2013). The effect of thyroid hormone disruption in the immature brain, however, is not well understood.

Although the causative mechanisms of ADHD remain unclear, a strong genetic component has been indicated in the etiology (Wood and Neale, 2010). A number of genetic association studies of ADHD have identified promising candidate genes related to both dopamine (DA) and 5-hydroxytryptamine (5-HT) systems (Oades, 2008). Genetic liability to ADHD is likely polygenic (Wood and Neale, 2010), but DA and 5-HT systems are believed to be involved in ADHD-like behavioral abnormality, as shown by phenotypic analysis of mice lacking DA transporter (DAT) (Gainetdinov et al., 1998; Zhuang et al., 2001), DA receptor 1 (Xu et al., 1994), or 5-HT receptor 1B (Brunner et al., 1999). Pharmacologic factors impacting DA/5-HT signaling during the neonatal period can also modulate adult monoaminergic function (Yu et al., 2014). For example, neonatal rats with 6-hydroxy-dopamine lesioning, a representative pharmacological model, exhibit spontaneous but age-limited locomotor hyperactivity (Avale et al., 2004; van der Kooij and Glennon, 2007). Such lesioning causes massive depletion of striatal DA and subsequent serotonergic hyperinnervation during the neonatal period; however, normalization induced by 5-HT depletion can reverse this hyperactivity (Avale et al., 2004). These observations implicate that DA/5-HT imbalance during the brain maturation process can cause the brain dysfunction (Gainetdinov et al., 1999; Avale et al., 2004; Oades, 2008).

Emerging evidence has demonstrated that the pathology of developmental mental disorders associates with aberrant epigenetic modifications (Graff et al., 2011). Rats with neonatal hypothyroidism are hyperactive and exhibit low histone acetylation in their developing brain (Lakshmy et al., 1999; Xu et al., 2007). Because thyroid hormone signaling is regulated by an epigenetic mechanism involving histone deacetylase (HDAC) (Ishizuka and Lazar, 2003; Vermeulen et al., 2004; You et al., 2010), a systemic profile of epigenetic states might reflect regional impairment caused by disruption of thyroid hormone signaling in the immature brain. The mammalian HDAC family consists of 4 protein groups: class I (HDAC1, -2, -3 and -8), class II (HDAC4, -5, -6, -7, -9 and -10), class III (sirtuins 1–7), and class IV with HDAC11 as the sole member. HDAC1, -2, and -3 are the major isoforms of class I and are mainly expressed in neurons (Broide et al., 2007). In this study, we selected several candidate regions that have dense monoaminergic projections in the mesocorticolimbic pathway. First, we assessed behavioral abnormalities in both TRα- and TRβ-mutant mice. Next, we measured tissue concentrations of DA, 5-HT, and their metabolites in several regions of developing brain. Then we determined protein expression of class I HDAC isoforms and acetylated histone H3 (acH3).

## Methods

### Animals

Littermates of TRα^*0/0*^ TRβ^+/+^ (TRα^*0/0*^) mice, TRα^+/+^TRβ^*−/−*^ (TRβ^*−/−*^) mice, and wild-type mice (WT) were generated from heterozygous dams and were used in behavioral and neurochemical experiments. TRα^*0/0*^ mice have a merit to minimalize the dominant negative effect caused by other TR subtypes (O’Shea and Williams, 2002). TRα^*0/0*^ and TRβ^*−/−*^mice were originally generated and propagated in the C57/BL6 background strain by Dr. J. Sumarut et al (Gauthier et al., 2001). Congenic mutants were gifted from Dr. K. Hashizume and backcrossed in the Laboratory Animal Center of Shiga University of Medical Science. It is possible that there is a gender difference due to crosstalk between thyroid hormones, estrogen and androgen; thereby, we used only male mice here. Mice were housed in a temperature-controlled environment with a 12-/12-hour-light–dark cycle and had access to food and water ad libitum. Comparisons were made among siblings within the same littermates. The experiments were conducted in accordance with the National Institutes of Health Guide for the Care and Use of Laboratory Animals. All experimental protocols were approved by the institutional animal care and use committee of the Shiga University of Medical Science.

### Behavioral Examination

Three-month-old male mice were used for an open-field test (OFT). The group compositions were WT (n=18), TRα^*0/0*^ (n=10), and TRβ^*−/−*^ (n=13) mice. Mice were kept in isolation for 4 weeks to acclimate to the test room and received handling until the start of experiment. Mice were individually placed in the center of a circular enclosure (75cm in diameter). The animals underwent 5 test sessions, each separated by 24 hours to ensure testing occurred at the same time each day. The activity data were collected and analyzed at a sampling rate of 0.1 second for 180 seconds by a video-tracking system (Muromachi Kikai Co. Ltd., Japan). In general, habituation in novel environments leads to a decline in horizontal exploratory activities over time either within a single test or multiple tests. Hyperactivity with reduced habituation is considered as a behavioral hallmark of ADHD-like abnormality (Zhuang et al., 2001). We thus performed the OFT with short exposures to novel circumstances under 80 lux to measure initial exploratory response without fear-related freezing. All 5 sessions were evaluated by travel distance (centimeters) and spent time (seconds). To discriminate emotional components in behaviors, activities including rearing, defecation/urination, and grooming were also quantified. The OFT arena was divided into 2 zones of equal area: central and peripheral. The total distances traveled as well as the time spent in these zones were measured.

### High Performance Liquid Chromatography (HPLC) Analysis

Male mice (n=6 per group) of 8 weeks of age were used. The brains were quickly removed and frozen with dry ice powder. HPLC analysis of DA and its metabolites in the brain regions was performed as previously described (Ookubo et al., 2008, 2013). The brains were coronally sectioned and then dissected into 7 regions, including the main olfactory bulb, caudate and putamen (CPu), nucleus accumbens (Acb), hippocampus (Hi), anterior cingulate cortex (Cg), amygdala, and dorsal raphe nucleus (DR), using corresponding coronal diagrams of the mouse brain atlas. The brain tissue samples of both hemispheres were sonicated in individual sterile tubes kept on ice using an ultrasonic homogenizer and then centrifuged; the resulting supernatants were placed in ice-cold 0.2M perchloric acid containing 100ng/mL isoproterenol as an internal standard. DA, 3, 4-dihydroxyphenylacetic acid (DOPAC), homovanillic acid (HVA), 5-HT, and 5-hydroxyindoleacetic acid (5-HIAA) were quantified by HPLC with an electrochemical detector (Eicom, Japan).

### Western Blotting of HDAC, acH3, and Tyrosine Hydroxylase (TH)

Male mice (n=6 per group) of 8 weeks of age were used. The brains were sectioned and dissected into 7 regions as described above. The frozen brain tissues were homogenized in HEPES-buffered sucrose (0.32M sucrose containing 4 μg/mL pepstatin, 5 μg/mL aprotinin, 20 μg/mL trypsin inhibitor, 4 μg/mL leupeptin, 0.2mM phenylmethanesulfonyl fluoride, 2mM EDTA, 2mM EGTA, and 20mM HEPES, pH 7.2) using an ultrasonic homogenizer as previously described (Ookubo et al., 2013). The homogenates were solubilized with LDS sample buffer (Invitrogen). Protein samples (10 μg) were loaded on 4% to 12% Bis-Tris Gel and transferred to polyvinylidene difluoride membranes with a semi-dry blotting system for 1 hour. The polyvinylidene difluoride membranes were incubated for 1 hour at room temperature with phosphate-buffered saline containing 0.1% Tween 20 and 0.5% skim milk, followed by overnight incubation at 4°C with the desired primary antibodies: anti-HDAC1 monoclonal antibody (1:1000, Santa Cruz Biotech), anti-HDAC2 polyclonal antibody (1:1000, Santa Cruz Biotech), anti-HDAC3 polyclonal antibody (1:1000), anti-acH3 (1:1000, Millipore, BRD), or anti-tyrosine hydroxylase (TH) monoclonal antibody (1:2000, Chemicon International Inc.). Monoclonal antibody against glyceraldehyde-3-phosphate dehydrogenase (1:4000, Sigma-Aldrich) was used to ensure equal sample loading. Membranes were washed 3 times for 10 minutes at room temperature and incubated with secondary antibodies in phosphate-buffered saline containing 0.1% Tween 20 containing 0.5% skim milk for 1 hour. Immunoreactive bands were visualized by enhanced chemiluminescent autoradiography (ECL Kit, Amersham) with Image Quant LAS-4000 (GE Healthcare). Optical densities were determined using a computerized image analysis system (MultiGauge, Fujifilm, Japan).

## Results

### Behavioral Analysis in the OFT

**Figure 1.**
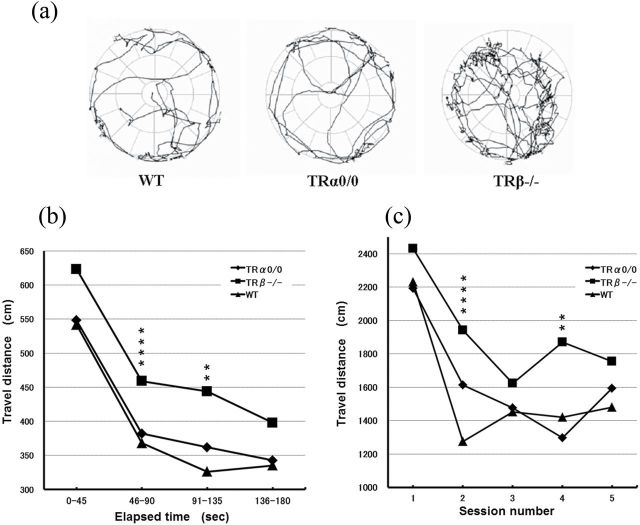
Open field analysis of expiratory activity in mice of TRα^*0/0*^, TRβ^*−/−*^, and wild-type (WT) mice at 3 months of age. Figure 1a shows typical tracks of each mice strain for 3 minutes on the fifth day of testing. TRβ^*−/−*^ mice exhibited hyperactivity. Figure 1b displays average distances of ambulation every 45-second bin for 3 minutes. Compared with WT mice, TRβ^*−/−*^ mice showed significant increased activity in the second and third quarter of a session. Each mouse strain nearly reached a steady state within 3 minutes of the start of an open-field test (OFT). Figure 1c displays average distances of ambulation in serial trials. TRβ^*−/−*^ mice displays to retain significant higher levels of activities in the second and fourth series of testing. Asterisks indicate significant differences from WT mice following ANOVA and Tukey’s HSD post hoc tests. Error bars represent ± SEM (**P < .01, ****P < .0001).

Locomotor activities were measured in WT, TRα^*0/0*^, and TRβ^*−/−*^ mice for 3 minutes per day for 5 days. No impairment of neuromotor performance, pathological stereotypy, or freezing was observed in any groups of congenic-conditioned mice at 3 months of age. WT, TRα^*0/0*^, and TRβ^*−/−*^ mice did not exhibit significant differences in mean velocity (WT: 21.2±0.2, TRα^*0/0*^: 21.4±0.5, TRβ^*−/−*^: 22.0±0.3, mean ± SEM, cm/s). Nonetheless, total distance travelled during all 5 sessions was significantly larger in TRβ^*−/−*^ mice than in WT mice, but TRα^*0/0*^ and WT mice showed similar levels (WT: 1571±62, TRα^*0/0*^: 1634±105, TRβ^*−/−*^: 1925±81, mean ± SEM, cm). Then, we focused on less habituation of the exploratory response to novel circumstances as a mark of ADHD-like hyperactivity. Figure 1a shows the typical tracks of each mouse strain on the fifth day. TRβ^*−/−*^ mice showed hyperactivity and tended to avoid making body contact with the wall while running along it. The repetition of, and time spent in, tests reduces the novelty of the testing field. The decline in exploratory activity for each group across time intervals and serial sessions was evaluated for intra- and inter-session habituation, respectively (Figure 1b-c). To compare inter-session habituation of exploratory activities, the data were binned into 45-second blocks for the statistical analysis of locomotor activity. Compared with WT mice, TRβ^*−/−*^ mice exhibited significantly prolonged locomotor responses in the middle of a session (Figure 1b). TRβ^*−/−*^ mice also displayed less habituation across the trial series (Figure 1c).

TRα^*0/0*^ mice exhibited a slight delay in entering novel areas from the starting point (a possible tendency of anxiety; data not shown), whereas TRβ^*−/−*^ mice had shorter latency. This suggests the possibility that TRα^*0/0*^ mice have an increase in anxiety as reported (Wilcoxon et al., 2007). Thus, we also assessed the impact of emotional factors on the locomotor activities and measured the number of times the animals reared and the ratio of time spent in the center vs the periphery of the open field. The sum of numbers of rearing behavior in all 5 of the sessions did not significantly differ among the groups (WT: 51±0.8, TRα^*0/0*^: 49±3.3, TRβ^*−/−*^: 43±0.7 times). The ratios of time spent in the center area vs the periphery also did not differ (WT: 31±3.5%, TRα^*0/0*^: 36±8.1%, TRβ^*−/−*^: 31±4.3%, percentage of time spent in the center area). These findings suggest that an emotional component, for example, fear, is not an important contributor to the observed differences in the horizontal exploratory activity. Collectively, TRβ^*−/−*^ mice displayed hyperactivity and less habituation in an initial response to novel inescapable circumstances, whereas TRα^*0/0*^ mice did not differ from WT mice.

### HPLC Analysis of DA and 5-HT Metabolism in Developing TR Mutant Mice

**Figure 2.**
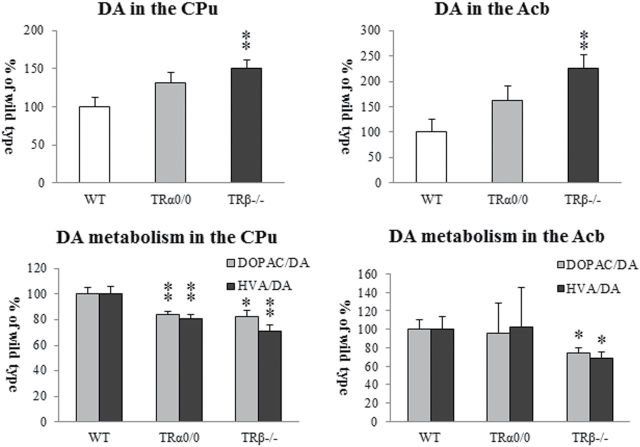
The high performance liquid chromatography (HPLC) analysis for dopamine (DA) contents and DA turnovers in the caudate and putamen (CPu) and nucleus accumbens (Acb) in TRα^*0/0*^, TRβ^*−/−*^, and wild-type (WT) mice at 8 weeks of age. Data were normalized for each protein level relative to WT mice. Asterisks indicate significant differences from WT mice following ANOVA and Tukey’s HSD post hoc tests. Error bars represent ± SEM (*P < .05, **P < .01).

We examined the levels of DA, 5-HT, and their metabolites in several brain regions richly innervated with DA and 5-HT fibers of 8-week-old mice. The results of the neurochemical analysis of DA and their metabolites are summarized in Table 1. TRβ^*−/−*^ mice showed significant changes in the CPu, Acb, and DR and high concentrations of tissue DA and low DA turnovers (Figure 2). Although TRβ^*−/−*^ mice showed slightly high DOPAC/HVA concentrations in these regions, the DA metabolic ratios significantly decreased, because of remarkably high tissue content of DA. Conversely, in the Cg of TRβ^*−/−*^ mice, the HVA level significantly decreased, but the ratio of HVA to DA appeared to be normal because of low tissue DA content. Similarly, the DA concentration in TRα^*0/0*^ mice showed an increase in the CPu, Acb, and DR but a decrease in the Cg. However, these changes did not reach statistical significance in TRα^*0/0*^ mice.

**Table 1.** HPLC Analysis for the Tissue Concentrations of DA, Its Metabolites, and Their Ratios to DA in TRα^*0/0*^, TRβ^*−/−*^ and WT Mice at 8 Weeks of Age.

DA	WT	TRα^*0/0*^	TRβ^*-/*-^
mOb	0.371±0.08	0.354±0.048	0.231±0.029
Cg	0.186±0.077	0.119±0.024	0.090±0.016
Cpu	10.859±1.880	14.224±1.488	16.261±1.198*
Acb	2.742±0.697	4.458±0.769	6.207±0.688**
Hi	0.094±0.018	0.306±0.113	0.331±0.145
Amy	0.574±0.098	0.783±0.175	0.725±0.147
DR	0.181±0.027	0.340±0.066	0.312±0.048
DOPAC	WT	TRα^*0/0*^	TRβ^*-/*-^
mOb	0.171±0.016	0.196±0.032	0.147±0.011
Cg	0.203±0.049	0.136±0.019	0.118±0.023
Cpu	0.666±0.054	0.759±0.065	0.812±0.055
Acb	0.469±0.048	0.466±0.043	0.604±0.060
Hi	0.146±0.011	0.173±0.012	0.176±0.014
Amy	0.247±0.023	0.230±0.017	0.248±0.015
DR	0.235±0.028	0.254±0.041	0.259±0.020
HVA	WT	TRα^*0/0*^	TRβ^*-/*-^
mOb	0.489±0.073	0.463±0.056	0.330±0.024
Cg	0.392±0.058	0.332±0.046	0.225±0.009**
Cpu	1.540±0.109	1.705±0.146	1.637±0.102
Acb	0.930±0.077	0.918±0.066	1.100±0.087
Hi	0.369±0.035	0.407±0.031	0.387±0.030
Amy	0.521±0.045	0.526±0.047	0.494±0.040
DR	0.477±0.049	0.572±0.051	0.534±0.032
DOPAC/DA	WT	TRα^*0/0*^	TRβ^*-/*-^
mOb	0.532±0.067	0.619±0.181	0.674±0.094
Cg	1.286±0.321	1.298±0.236	1.329±0.109
Cpu	0.065±0.003	0.055±0.002*	0.054±0.003*
Acb	0.154±0.016	0.146±0.051	0.115±0.009*
Hi	1.841±0.189	1.370±0.259	1.588±0.331
Amy	0.453±0.048	0.387±0.073	0.409±0.067
DR	1.394±0.147	0.793±0.094**	0.873±0.086**
HVA/DA	WT	TRα^*0/0*^	TRβ^*-/*-^
mOb	1.472±0.215	1.474±0.391	1.474±0.088
Cg	2.747±0.638	3.072±0.482	2.717±0.320
Cpu	0.152±0.009	0.123±0.005*	0.107±0.008**
Acb	0.302±0.042	0.310±0.129	0.207±0.022*
Hi	4.927±0.763	3.220±0.607	3.547±0.734
Amy	0.987±0.119	0.815±0.125	0.763±0.085
DR	2.998±0.515	1.850±0.254*	1.813±0.178*

Mean concentrations and SDs are expressed in µg/g tissue weight. Asterisks indicate significant differences from wild-type (WT) mice following ANOVA and Tukey’s HSD post hoc tests. Error bars represent ± SEM (*P < .05, **P < .01).

**Figure 3.**
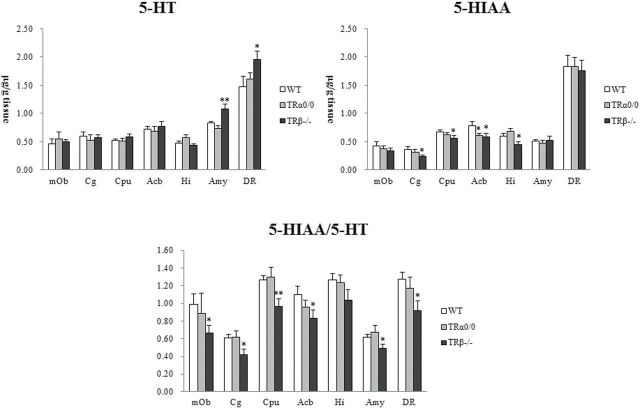
The high performance liquid chromatography (HPLC) analysis for tissue concentrations of 5-hydroxytryptamine (5-HT), 5-hydroxyindoleacetic acid (5-HIAA), and 5-HT metabolism ratios in TRα^*0/0*^, TRβ^*−/−*^, and wild-type (WT) mice at 8 weeks of age. The 5-HT turnovers in most brain regions of TRβ^*−/−*^ mice were significant lower than those in WT mice. Mean concentrations and standard deviations are expressed in µg/g tissue weight. Asterisks indicate significant differences from WT mice following ANOVA and Tukey’s HSD post hoc tests. Error bars represent ± SEM (*P < .05, **P < .01).

The HPLC analysis of 5-HT and its metabolites is graphically shown in Figure 3. In the amygdala and DR, 5-HT concentration significantly increased in TRβ^*−/−*^ mice but not in TRα^*0/0*^ mice. In the Cg, CPu, Acb, and Hi, tissue 5-HIAA levels in TRβ^*−/−*^ mice significantly decreased, while those in TRα^0/0^ mice were almost normal. Despite such differences in detail, the 5-HIAA/5-HT ratio, a 5-HT turnover index, was significantly lower in all brain regions of TRβ^*−/−*^ mice compared with WT mice, in which the ratios ranged from 0.42±0.06 (Cg) to 1.04±0.11 (Hi). The 5-HIAA/5-HT ratios of TRα^*0/0*^ mice remained at normal levels in all the brain regions. These findings suggest that 5-HT function is severely altered in TRβ^*−/−*^ mice, but not in TRα^*0/0*^ mice, at 8 weeks of age.

### Protein Expression of TH in the CPu and Acb

**Figure 4.**
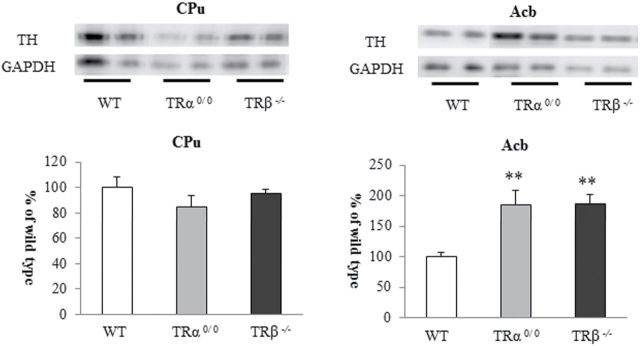
The expression of tyrosine hydroxylase (TH) proteins in the caudate and putamen (CPu) and nucleus accumbens (Acb) in TRα^*0/0*^, TRβ^*−/−*^, and wild-type (WT) mice at 8 weeks of age. Lanes in the upper part of the panels show a part of the blots of Western analysis. Asterisks indicate significant differences from WT mice following ANOVA and Tukey’s HSD post hoc tests. Error bars represent ± SEM (**P < .01).

The CPu and Acb receive dopaminergic projections most prominently in the brain. Tissue protein levels of TH, the rate-limiting enzyme in DA synthesis, can be affected by changes in synaptic modification such as dopaminergic hyperinnervation. TH expression remained unchanged in the CPu, whereas it was significantly elevated in the Acb of both TRα^*0/0*^ and TRβ^*−/−*^ mice (Figure 4). It raised the possibility that TR dysfunction could cause minor structural changes in the Acb (supplementary Figure S1). However, TH expressions almost equally increased in TRα^*0/0*^ and TRβ^*−/−*^ mice. Accordingly, the difference in DA metabolism between TRα^*0/0*^ and TRβ^*−/−*^ mice may imply the involvement of other regulatory mechanisms.

### Protein Expressions of acH3 and Class I HDAC Isoforms

**Figure 5.**
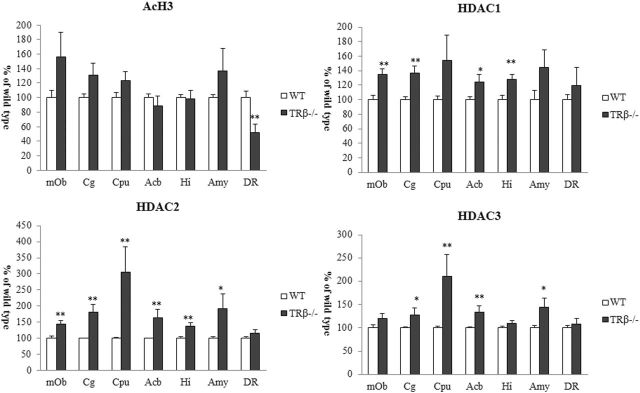
The expression of acetylated histone H3 (acH3), histone deacetylase (HDAC)1, HDAC2, and HADC3 proteins in TRβ^*−/−*^ and wild-type (WT) mice at 8 weeks of age. Asterisks indicate significant differences from WT mice following ANOVA and Tukey’s HSD post hoc tests. Error bars represent ± SEM (*P < .05, **P < .01).

Expression of each protein was determined in mice at 8 weeks of age. TRβ^*−/−*^ mice showed restricted change of histone acetylation (Figure 5). Notably, there was a significant decrease of acH3 in the serotonergic DR of TRβ^*−/−*^ mice but not in TRα^*0/0*^ mice (data not shown). HDAC1 expression tended to increase in the main olfactory bulb, Cg, Acb, and Hi of TRβ^*−/−*^ mice. The HDAC2 and HDAC3 expressions had a similar profile and displayed significant increases in most brain regions. HDAC2/3 expression was higher overall than HDAC1 expression. In particular, the expression of HDAC2/HDAC3, but not HDAC1, significantly increased in the CPu of TRβ^*−/−*^ mice. Meanwhile, modifications of the histone acetylation and HDAC1-3 protein levels were unlikely associated with each other in the corresponding region, presuming disrupted epigenetic equilibrium between histone acetylation and HDAC proteins.

## Discussion

Some kinds of TRβ transgenic mice have been demonstrated to be hyperactive phenotypes characterized by less habituation or a paradoxical response to methylphenidate treatment (O’Shea and Williams, 2002; Siesser et al., 2006). In contrast, TRα^*0/0*^ mice show mild hypoactivity and an increase in anxiety (Wilcoxon et al., 2007). Similarly, our results in the OFT demonstrated that TRβ^*−/−*^ mice showed high exploratory activity with reduced habituation, while TRα^*0/0*^ mice showed normal exploratory activity and a possible increase in anxiety. In biochemical profiles in the candidate regions for the behavioral abnormality, the TRβ deficiency caused both DA and 5-HT dysfunction and epigenetic disruption during the growth period.

In genetic models of ADHD, increased striatal DA turnover has often been observed. DAT knockout/knockdown mice have the highest degree of validity as an ADHD model with striatal hyperdopaminergic characteristics (Zhuang et al., 2001; van der Kooij and Glennon, 2007). In DAT knockout/knockdown mice, DA clearance from the synaptic cleft is greatly reduced, resulting in a prolonged and high concentration of extracellular DA, a high concentration of tissue DOPAC, downregulation of DA receptors, and compensatory low levels of tissue DA stores and TH expression (Gainetdinov et al., 1998; Zhuang et al., 2001). Spontaneous locomotor hyperactivity under normal circumstances is related to increased dopaminergic tone in the CPu, which is a consequence of increased DA release/turnover (Gainetdinov et al., 1998). On the other hand, ADHD-like abnormalities can also be observed in hypodopaminergic models (van der Kooij and Glennon, 2007). Mice lacking a DA receptor 1-signaling molecule such as DARPP-32 and p35 show a low DOPAC/DA ratio but compensatory high levels of tissue DA stores and TH expression (Drerup et al., 2010; Krapacher et al., 2010; Napolitano et al., 2010). In the present study, TRβ^*−/−*^ mice appeared to be a model of the striatal hypodopaminergic state, because significantly low DA turnover and a high level of tissue DA content were observed in the CPu and Acb of TRβ^*−/−*^ mice at 8 weeks of age. However, no significant differences in monoaminergic metabolism were observed among the groups of adult mice of the same strains (M. Ookubo and H. Kanai, unpublished observations).

Dramatic cytoarchitectural maturation occurs within the first month after birth in the normal CPu; although thyroid hormone deficiency does not entirely prevent the development of the CPu, this deficiency leads to a fairly extensive though critically incomplete degree of maturation, including a decrease in the number of synaptic contacts (Lu and Brown, 1977a, 1977b). The CPu has the highest expression of both TRα and TRβ in rats throughout the prenatal and neonatal periods but shows less expression of TRβ by adulthood (Bradley et al., 1989, 1992). In addition, thyroid hormone dependency of neurite outgrowth for 5-HT and noradrenaline- and DA-containing neurons has been reported in an ex vivo study (Granholm et al., 1984). Therefore, TR mutant mice might develop neural disorganization due to a distinct growth inhibition in the basal ganglia, although detailed information on the synaptic functions has yet to be determined.

We also found that TRβ^*−/−*^ mice, but not TR^0/0^ mice, exhibited a normal or high 5-HT content and overall low serotonergic metabolism in the mesolimbic regions. In addition, the serotonergic DR of TRβ^*−/−*^ mice showed a significantly decrease in histone acetylation. Similarly, hyperactive phenotypes like ADHD with low 5-HT metabolism have been found in mice lacking various genes (Chiavegatto et al., 2001; Hashimoto et al., 2001; Trent et al., 2012). Delay aversion and impulsive choices like ADHD symptoms are associated with dysfunction in the dopaminergic reward signal, and this reward processing is anatomically and physiologically related to the 5-HT system (Winstanley et al., 2006; Miyazaki et al., 2012; Nakamura, 2013). In animal models, systemic 5-HT depletion causes not only this inhibitory-based executive deficit but also exploratory hyperactivity (Breese et al., 1975; Dringenberg et al., 1995; Mobini et al., 2000). Thus, the hyposerotonergic state in the reward system might contribute to hyperactivity in TRβ^−/−^ mice.

Lastly, we are the first study to demonstrate epigenetic alterations in TRβ^−/−^ mice; this could be said to be unique characteristics of this ADHD model. The low acH3 in the serotonergic DR and the high expression of HDAC2/3 proteins in the mesolimbic DA system may underlie the functional changes in monoaminergic tone. In contrast, the changes in HDAC expression levels in TR^*0/0*^ mice were mostly smaller than those in TRβ^−/−^ mice (M. Ookubo and H. Kanai, unpublished observations). Sustained changes in HDAC activity and histone modification can stably modify cell activity ex vivo (Kanai et al., 2004). Recently, epigenetic changes in a specific brain region have been implicated in persistent behavioral changes, including drug dependence and memory formation (Morris et al., 2010; Malvaez et al., 2011; Ookubo et al., 2013). Indeed, manipulation of the expression of HDAC1, -2, and -3 differentially modulates synaptic plasticity such as memory facilitation in the Hi of mice (Akhtar et al., 2009; Morris et al., 2010; McQuown and Wood, 2011).

Among HDAC isoforms, HDAC3 is the most abundant isoform in neurons (Broide et al., 2007) and forms a tight complex with NcoR (nuclear receptor co-repressor) and SMRT (silencing mediator for retinoid and thyroid hormone receptor); this complex binds unliganded TR (Ishizuka and Lazar, 2003; Vermeulen et al., 2004; You et al., 2010). According to transfection studies, knockdown of HDAC3 or prevention of the interaction between NcoR and HDAC3 markedly decreases the magnitude of gene repression by TR, while knockdown of HDAC1 or HDAC2 has more modest and partly nonspecific effects (Ishizuka and Lazar, 2003). Conversely, class II HDAC isoforms are solely inactive but can bind to HDAC3/NcoR complexes and facilitate the action of HDAC3 (Yang and Gregoire, 2005). Although further research is needed to elucidate various roles of HDAC isoforms in the developing brain, our results show the possibility that disruption of epigenetic modulators also contributes to develop behavioral abnormality.

## Statement of Interest

None.

## Supplementary Material

supplementary Figure S1

## References

[CIT0001] Akhtar MW Raingo J Nelson ED Montgomery RL Olson EN Kavalali ET Monteggia LM (2009) Histone deacetylases 1 and 2 form a developmental switch that controls excitatory synapse maturation and function. J Neurosci 29:8288–8297. 19553468 10.1523/JNEUROSCI.0097-09.2009 PMC2895817

[CIT0002] Avale ME Nemirovsky SI Raisman-Vozari R Rubinstein M (2004) Elevated serotonin is involved in hyperactivity but not in the paradoxical effect of amphetamine in mice neonatally lesioned with 6-hydroxydopamine. J Neurosci Res 78:289–296. 15378510 10.1002/jnr.20245

[CIT0003] Bradley DJ Young WS III Weinberger C (1989) Differential expression of alpha and beta thyroid hormone receptor genes in rat brain and pituitary. Proc Natl Acad Sci U S A 86:7250–7254. 2780568 10.1073/pnas.86.18.7250 PMC298035

[CIT0004] Bradley DJ Towle HC Young WS III (1992) Spatial and temporal expression of alpha- and beta-thyroid hormone receptor mRNAs, including the beta 2-subtype, in the developing mammalian nervous system. J Neurosci 12:2288–2302. 1607941 10.1523/JNEUROSCI.12-06-02288.1992 PMC6575910

[CIT0005] Breese GR Cooper BR Hollister AS (1975) Involvement of brain monoamines in the stimulant and paradoxical inhibitory effects of methylphenidate. Psychopharmacologia 44:5–10. 128026 10.1007/bf00421175 PMC2904632

[CIT0006] Broide RS Redwine JM Aftahi N Young W Bloom FE Winrow CJ (2007) Distribution of histone deacetylases 1–11 in the rat brain. J Mol Neurosci 31:47–58. 17416969 10.1007/BF02686117

[CIT0007] Brucker-Davis F Skarulis MC Grace MB Benichou J Hauser P Wiggs E Weintraub BD (1995) Genetic and clinical features of 42 kindreds with resistance to thyroid hormone. The National Institutes of Health Prospective Study. Ann Intern Med 123:572–583. 7677297 10.7326/0003-4819-123-8-199510150-00002

[CIT0008] Brunner D Buhot MC Hen R Hofer M (1999) Anxiety, motor activation, and maternal-infant interactions in 5HT1B knockout mice. Behav Neurosci 113:587–601. 10443785 10.1037//0735-7044.113.3.587

[CIT0009] Cheng SY Leonard JL Davis PJ (2010) Molecular aspects of thyroid hormone actions. Endocr Rev 31:139–170. 20051527 10.1210/er.2009-0007 PMC2852208

[CIT0010] Chiavegatto S Dawson VL Mamounas LA Koliatsos VE Dawson TM Nelson RJ (2001) Brain serotonin dysfunction accounts for aggression in male mice lacking neuronal nitric oxide synthase. Proc Natl Acad Sci U S A 98:1277–1281. 11158630 10.1073/pnas.031487198 PMC14745

[CIT0011] Drerup JM Hayashi K Cui H Mettlach GL Long MA Marvin M Sun X Goldberg MS Lutter M Bibb JA (2010) Attention-deficit/hyperactivity phenotype in mice lacking the cyclin-dependent kinase 5 cofactor p35. Biol Psychiatry 68:1163–1171. 20832057 10.1016/j.biopsych.2010.07.016 PMC2997929

[CIT0012] Dringenberg HC Hargreaves EL Baker GB Cooley RK Vanderwolf CH (1995) p-Chlorophenylalanine-induced serotonin depletion: reduction in exploratory locomotion but no obvious sensory-motor deficits. Behav Brain Res 68:229–237. 7544584 10.1016/0166-4328(94)00174-e

[CIT0013] Gainetdinov RR Jones SR Fumagalli F Wightman RM Caron MG (1998) Re-evaluation of the role of the dopamine transporter in dopamine system homeostasis. Brain Res Brain Res Rev 26:148–153. 9651511 10.1016/s0165-0173(97)00063-5

[CIT0014] Gainetdinov RR Wetsel WC Jones SR Levin ED Jaber M Caron MG (1999) Role of serotonin in the paradoxical calming effect of psychostimulants on hyperactivity. Science 283:397–401. 9888856 10.1126/science.283.5400.397

[CIT0015] Gauthier K Plateroti M Harvey CB Williams GR Weiss RE Refetoff S Willott JF Sundin V Roux JP Malaval L Hara M Samarut J Chassande O (2001) Genetic analysis reveals different functions for the products of the thyroid hormone receptor alpha locus. Mol Cell Biol 21:4748–4760. 11416150 10.1128/MCB.21.14.4748-4760.2001 PMC87157

[CIT0016] Graff J Kim D Dobbin MM Tsai LH (2011) Epigenetic regulation of gene expression in physiological and pathological brain processes. Physiol Rev 91:603–649. 21527733 10.1152/physrev.00012.2010

[CIT0017] Granholm AC Siegel RA Seiger A (1984) Thyroid hormone dependency of the developing dorsal raphe nucleus but not the superior cervical ganglion. Evidence from intraocular grafting experiments. Int J Dev Neurosci 2:337–345. 24874144 10.1016/0736-5748(84)90070-4

[CIT0018] Harley KG Gunier RB Kogut K Johnson C Bradman A Calafat AM Eskenazi B (2013) Prenatal and early childhood bisphenol A concentrations and behavior in school-aged children. Environ Res 126:43–50. 23870093 10.1016/j.envres.2013.06.004 PMC3805756

[CIT0019] Hashimoto H Shintani N Tanaka K Mori W Hirose M Matsuda T Sakaue M Miyazaki J Niwa H Tashiro F Yamamoto K Koga K Tomimoto S Kunugi A Suetake S Baba A (2001) Altered psychomotor behaviors in mice lacking pituitary adenylate cyclase-activating polypeptide (PACAP). Proc Natl Acad Sci U S A 98:13355–13360. 11687615 10.1073/pnas.231094498 PMC60875

[CIT0020] Hauser P Zametkin AJ Martinez P Vitiello B Matochik JA Mixson AJ Weintraub BD (1993) Attention deficit-hyperactivity disorder in people with generalized resistance to thyroid hormone. N Engl J Med 328:997–1001. 8450877 10.1056/NEJM199304083281403

[CIT0021] Ishizuka T Lazar MA (2003) The N-CoR/histone deacetylase 3 complex is required for repression by thyroid hormone receptor. Mol Cell Biol 23:5122–5131. 12861000 10.1128/MCB.23.15.5122-5131.2003 PMC165720

[CIT0022] Kanai H Sawa A Chen RW Leeds P Chuang DM (2004) Valproic acid inhibits histone deacetylase activity and suppresses excitotoxicity-induced GAPDH nuclear accumulation and apoptotic death in neurons. Pharmacogenomics J 4:336–344. 15289798 10.1038/sj.tpj.6500269

[CIT0023] Krapacher FA Mlewski EC Ferreras S Pisano V Paolorossi M Hansen C Paglini G (2010) Mice lacking p35 display hyperactivity and paradoxical response to psychostimulants. J Neurochem 114:203–214. 20403084 10.1111/j.1471-4159.2010.06748.x

[CIT0024] Lakshmy R Khurana ML Das BC Shah P Ammini AC (1999) Effect of PTU treatment on histone acetylation pattern in the developing rat brain. Endocr Res 25:77–85. 10098595 10.1080/07435809909066131

[CIT0025] Lu EJ Brown WJ(1977a) The developing caudate nucleus in the euthyroid and hypothyroid rat. J Comp Neurol 171:261–284. 833351 10.1002/cne.901710209

[CIT0026] Lu EJ Brown WJ(1977b) An electron microscopic study of the developing caudate nucleus in euthyroid and hypothyroid states. Anat Embryol (Berl) 150:335–364. 869226 10.1007/BF00318351

[CIT0027] Malvaez M Mhillaj E Matheos DP Palmery M Wood MA (2011) CBP in the nucleus accumbens regulates cocaine-induced histone acetylation and is critical for cocaine-associated behaviors. J Neurosci 31:16941–16948. 22114264 10.1523/JNEUROSCI.2747-11.2011 PMC3235434

[CIT0028] McQuown SC Wood MA (2011) HDAC3 and the molecular brake pad hypothesis. Neurobiol Learn Mem 96:27–34. 21521655 10.1016/j.nlm.2011.04.005 PMC3111848

[CIT0029] Miyazaki KW Miyazaki K Doya K (2012) Activation of dorsal raphe serotonin neurons is necessary for waiting for delayed rewards. J Neurosci 32:10451–10457. 22855794 10.1523/JNEUROSCI.0915-12.2012 PMC6621383

[CIT0030] Mobini S Chiang TJ Al-Ruwaitea AS Ho MY Bradshaw CM Szabadi E (2000) Effect of central 5-hydroxytryptamine depletion on inter-temporal choice: a quantitative analysis. Psychopharmacology (Berl) 149:313–318. 10823413 10.1007/s002130000385

[CIT0031] Morris MJ Karra AS Monteggia LM (2010) Histone deacetylases govern cellular mechanisms underlying behavioral and synaptic plasticity in the developing and adult brain. Behav Pharmacol 21:409–419. 20555253 10.1097/FBP.0b013e32833c20c0 PMC2923662

[CIT0032] Nakamura K (2013) The role of the dorsal raphe nucleus in reward-seeking behavior. Front Integr Neurosci 7:60. 23986662 10.3389/fnint.2013.00060 PMC3753458

[CIT0033] Napolitano F Bonito-Oliva A Federici M Carta M Errico F Magara S Martella G Nistico R Centonze D Pisani A Gu HH Mercuri NB Usiello A (2010) Role of aberrant striatal dopamine D1 receptor/cAMP/protein kinase A/DARPP32 signaling in the paradoxical calming effect of amphetamine. J Neurosci 30:11043–11056. 20720111 10.1523/JNEUROSCI.1682-10.2010 PMC6633484

[CIT0034] O’Shea PJ Williams GR (2002) Insight into the physiological actions of thyroid hormone receptors from genetically modified mice. J Endocrinol 175:553–570. 12475367 10.1677/joe.0.1750553

[CIT0035] Oades RD (2008) Dopamine-serotonin interactions in attention-deficit hyperactivity disorder (ADHD). Prog Brain Res 172:543–565. 18772050 10.1016/S0079-6123(08)00926-6

[CIT0036] Ookubo M Yokoyama H Takagi S Kato H Araki T (2008) Effects of estrogens on striatal damage after 1-methyl-4-phenyl-1,2,3,6-tetrahydropyridine (MPTP) neurotoxicity in male and female mice. Mol Cell Endocrinol 296:87–93. 18755240 10.1016/j.mce.2008.07.019

[CIT0037] Ookubo M Kanai H Aoki H Yamada N (2013) Antidepressants and mood stabilizers effects on histone deacetylase expression in C57BL/6 mice: brain region specific changes. J Psychiatr Res 47:1204–1214. 23777937 10.1016/j.jpsychires.2013.05.028

[CIT0038] Schoenmakers N Moran C Peeters RP Visser T Gurnell M Chatterjee K (2013) Resistance to thyroid hormone mediated by defective thyroid hormone receptor alpha. Biochim Biophys Acta 1830:4004–4008. 23528896 10.1016/j.bbagen.2013.03.018

[CIT0039] Siesser WB Zhao J Miller LR Cheng SY McDonald MP (2006) Transgenic mice expressing a human mutant beta1 thyroid receptor are hyperactive, impulsive, and inattentive. Genes Brain Behav 5:282–297. 16594981 10.1111/j.1601-183X.2005.00161.x

[CIT0040] Trent S Cassano T Bedse G Ojarikre OA Humby T Davies W (2012) Altered serotonergic function may partially account for behavioral endophenotypes in steroid sulfatase-deficient mice. Neuropsychopharmacology 37:1267–1274. 22189290 10.1038/npp.2011.314 PMC3306888

[CIT0041] van der Kooij MA Glennon JC (2007) Animal models concerning the role of dopamine in attention-deficit hyperactivity disorder. Neurosci Biobehav Rev 31:597–618. 17316796 10.1016/j.neubiorev.2006.12.002

[CIT0042] Vermeulen M Carrozza MJ Lasonder E Workman JL Logie C Stunnenberg HG (2004) In vitro targeting reveals intrinsic histone tail specificity of the Sin3/histone deacetylase and N-CoR/SMRT corepressor complexes. Mol Cell Biol 24:2364–2372. 14993276 10.1128/MCB.24.6.2364-2372.2004 PMC355843

[CIT0043] Vermiglio F Lo Presti VP Moleti M Sidoti M Tortorella G Scaffidi G Castagna MG Mattina F Violi MA Crisa A Artemisia A Trimarchi F (2004) Attention deficit and hyperactivity disorders in the offspring of mothers exposed to mild-moderate iodine deficiency: a possible novel iodine deficiency disorder in developed countries. J Clin Endocrinol Metab 89:6054–6060. 15579758 10.1210/jc.2004-0571

[CIT0044] Weyandt LL Dupaul GJ (2008) ADHD in college students: Developmental findings. Dev Disabil Res Rev 14:311–319. 19072759 10.1002/ddrr.38

[CIT0045] Wilcoxon JS Nadolski GJ Samarut J Chassande O Redei EE (2007) Behavioral inhibition and impaired spatial learning and memory in hypothyroid mice lacking thyroid hormone receptor alpha. Behav Brain Res 177:109–116. 17129617 10.1016/j.bbr.2006.10.030 PMC1819397

[CIT0046] Winstanley CA Eagle DM Robbins TW (2006) Behavioral models of impulsivity in relation to ADHD: translation between clinical and preclinical studies. Clin Psychol Rev 26:379–395. 16504359 10.1016/j.cpr.2006.01.001 PMC1892795

[CIT0047] Wood AC Neale MC (2010) Twin studies and their implications for molecular genetic studies: endophenotypes integrate quantitative and molecular genetics in ADHD research. J Am Acad Child Adolesc Psychiatry 49:874–883. 20732624 10.1016/j.jaac.2010.06.006 PMC3148177

[CIT0048] Xu M Moratalla R Gold LH Hiroi N Koob GF Graybiel AM Tonegawa S (1994) Dopamine D1 receptor mutant mice are deficient in striatal expression of dynorphin and in dopamine-mediated behavioral responses. Cell 79:729–742. 7954836 10.1016/0092-8674(94)90557-6

[CIT0049] Xu X Liu Y Sadamatsu M Tsutsumi S Akaike M Ushijima H Kato N (2007) Perinatal bisphenol A affects the behavior and SRC-1 expression of male pups but does not influence on the thyroid hormone receptors and its responsive gene. Neurosci Res 58:149–155. 17412439 10.1016/j.neures.2007.02.011

[CIT0050] Yang XJ Gregoire S (2005) Class II histone deacetylases: from sequence to function, regulation, and clinical implication. Mol Cell Biol 25:2873–2884. 15798178 10.1128/MCB.25.8.2873-2884.2005 PMC1069616

[CIT0051] You SH Liao X Weiss RE Lazar MA (2010) The interaction between nuclear receptor corepressor and histone deacetylase 3 regulates both positive and negative thyroid hormone action in vivo. Mol Endocrinol 24:1359–1367. 20427468 10.1210/me.2009-0501 PMC2903906

[CIT0052] Yu Q Teixeira CM Mahadevia D Huang Y Balsam D Mann JJ Gingrich JA Ansorge MS (2014) Dopamine and serotonin signaling during two sensitive developmental periods differentially impact adult aggressive and affective behaviors in mice. Mol Psychiatry 19:688–698. 24589889 10.1038/mp.2014.10 PMC4311886

[CIT0053] Zhuang X Oosting RS Jones SR Gainetdinov RR Miller GW Caron MG Hen R (2001) Hyperactivity and impaired response habituation in hyperdopaminergic mice. Proc Natl Acad Sci U S A 98:1982–1987. 11172062 10.1073/pnas.98.4.1982 PMC29368

