# Supplementary material for: Aberrant Monoaminergic System in Thyroid Hormone Receptor-β Deficient Mice as a Model of Attention-Deficit/Hyperactivity Disorder
Source: Int J Neuropsychopharmacol. 2015 Mar 20;18(7):pyv004. doi: 10.1093/ijnp/pyv004 (PMC4540106; doi:10.1093/ijnp/pyv004)
Supplement: supplementary Figure S1 [file Supplementary_Fig.docx]

**Supplementary Fig. S1**

The expression of GFAP protein in TRβ*^−/−^* and WT mice at 8 weeks of age. An increase in GFAP expression is an indicator for minor histopathological changes, including neuronal damage and glial activation. Compared to WT mice, GFAP expression was significantly increased in the Acb and Hi of TRα*^0/0^* mice. TRβ*^−/−^* mice also showed a significantly increase in GFAP expression in the CPu, Acb and Hi, but not in the DR and Amy. Error bars represent ± S.E.M. (**p* < 0.05, ***p* < 0.01).
