# Supplementary material for: Aberrant Monoaminergic System in Thyroid Hormone Receptor-β Deficient Mice as a Model of Attention-Deficit/Hyperactivity Disorder
Source: Int J Neuropsychopharmacol. 2015 Mar 20;18(7):pyv004. doi: 10.1093/ijnp/pyv004 (PMC4540106; doi:10.1093/ijnp/pyv004)
Supplement: supplementary Figure S1 [file Fig.S1_pptx.ppt]

## Slide 1
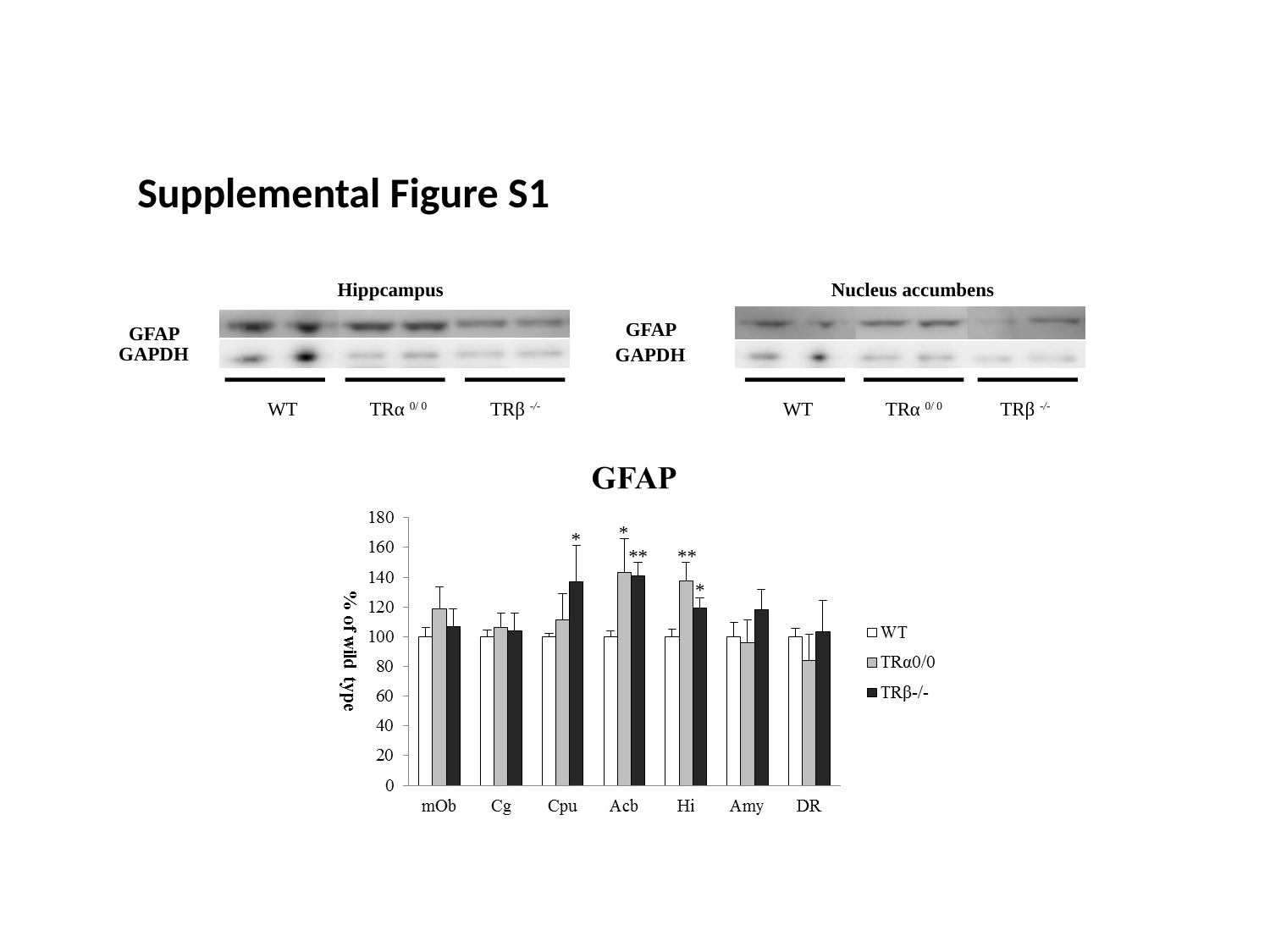

Supplemental Figure S1
Hippcampus
Nucleus accumbens
GFAP
GFAP
GAPDH
GAPDH
WT
TRα 0/ 0
TRβ -/-
WT
TRα 0/ 0
TRβ -/-
*
*
**
**
*
